# G-quadruplex structure in plants and insects and potential applications in pest control

**DOI:** 10.1007/s44297-025-00047-2

**Published:** 2025-04-02

**Authors:** Xiaojuan Zhang, Lijun Xiang, Jin Li, Qili Feng, Kangkang Niu

**Affiliations:** Guangdong Provincial Key Laboratory of Insect Developmental Biology and Applied Technology, Guangzhou Key Laboratory of Insect Development Regulation and Application Research, Institute of Insect Science and Technology, School of Life Sciences, South China Normal University, Guangzhou, 510631 China

**Keywords:** G-quadruplex, DNA advanced structure, Insect, Plant, Pest control

## Abstract

The guanine-enriched regions of nucleic acids can adopt four-stranded G-quadruplex structures (G4s). Considerable evidence reveals that predicted G4-forming sequences prevalently exist in the genomes of various organisms. The abundant G4 formation linked G4s to fundamental biological processes such as transcription, replication, translation and telomere protection. G4s are also known to be closely associated with many diseases, especially cancer. However, an increasing number of studies have investigated the critical roles of G4s in the development of plants and insects. In this review, we discuss the characteristics of the distribution and regulation of G4s and their biological roles in plants and insects, as well as the potential application of G4s as molecular targets in pest control.

## Introduction

**Fig. 1:**
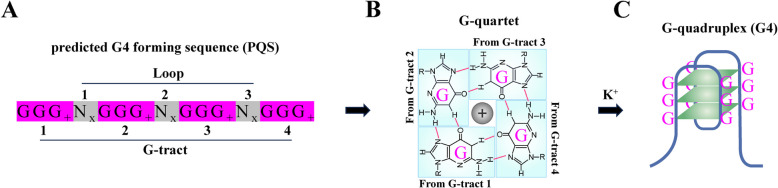
The structure of G4s. **A** The predicted G4-forming sequence (PQS). N: A, T, C or G; x: the number of nucleotides in the loop. On the basis of the G_3+_N_x_G_3+_N_x_G_3+_N_x_G_3+_ motif, PQS prediction can be performed via several quadparser algorithms, such as pqsfinder [6] and the QGRS Mapper [7]. **B** G-quartet formed by four Gs. **C** The G-quadruplex structure formed by three G-quartets

Double-stranded DNA molecules usually exist in the form of linear B-form double-helix structures [1]. However, DNA sequences can also form non-B DNA structures, such as hairpin, triplex DNA and four-stranded G-quadruplex (G4) structures, among which G4 has been widely studied [2]. A G4 consists of two or more stacked G-quartets, which are assembled from four guanines by Hoogsteen hydrogen bonding [3]. The Hoogsteen hydrogen bond, which was first described by the biochemist Arthur Hoogsteen in 1963 [4], is a specific type of hydrogen bonding that occurs in nucleic acids, particularly in DNA. The donor and acceptor positions are altered in Hoogsteen hydrogen bonds, which is different from the conventional Watson‒Crick base pairs. For example, the N7 position of guanine (instead of the usual N9 position) is involved in forming hydrogen bonds. Hoogsteen hydrogen bonds typically occur in higher-order DNA structures, such as G-quadruplexes and triplex DNA [3]. Monovalent cations, such as K^+^, Na^+^ and NH_4_^+^, can stabilize G4s [5]. On the basis of the classical characteristics of predicted G4-forming sequences (PQSs, Fig. 1A) [6, 7], PQSs have been identified to be prevalent in the genomes of different species, and the distribution of PQSs is not random but highly and specifically enriched in regulatory regions, such as replication origins, promoters, telomeres and untranslated regions (UTRs) [8]. The whole-genome-wide prediction of PQSs is a fast and effective method for investigating the distribution of G4s, and it could be used for a variety of purposes. In the initial stage of research, PQS prediction could act as a guide for researchers to investigate interesting issues, such as whether target genes are regulated by G4s and what types of genes that participate in critical regulatory processes contain G4s in their regulatory regions. In addition to gene regulation, PQS prediction could also be beneficial for other studies associated with G4s, such as single nucleotide polymorphisms and epigenetic modifications. With the help of the acquisition of sequence information in different genomes, PQS prediction could favor the identification of G4 distributions throughout genomes, and the detailed location of G4s could reveal the possible functions of G4s in critical processes. With the use of G4-specific antibodies and fluorescent probes, G4s have been directly or indirectly detected under cellular conditions [9, 10]. In addition, the identification of many G4-specific binding proteins also implies the presence of G4s in vivo [11]. Studies have revealed that G4s are closely related to many regulatory roles in cells, such as DNA replication, gene transcription and genome stability. Notably, experimental evidence indicates that G4s play crucial regulatory roles in the occurrence and development of human diseases, especially cancer, making G4s an emerging therapeutic target [12]. As interest in G4s in human diseases continues to grow, significant progress in G4s has also been made in other fields, providing novel insights into understanding and elucidating the biological functions of G4s. In this review, we summarize recent progress in the study of G4s and their functions in plants and insects and discuss the potential of G4s as molecular targets for the control of agricultural pests.

### The structure and biological functions of G4s

Since the discovery of the DNA structure in 1953, DNA has been widely believed to exist as a typical double-strand helical structure with strict base pairings between adenine (A) and thymine (T) and between cytosine (C) and guanine (G) [1]. However, increasing experimental evidence has revealed that in addition to this canonical structure, DNA molecules can form various noncanonical advanced structures, such as hairpin DNA, cruciform DNA, triplex stranded DNA and quadruple DNA [2], in specific regions and under certain cellular conditions. Among these advanced DNA structures, G4 is thermodynamically stable and has been the most extensively studied recently. In 1962, Gellert and colleagues demonstrated that guanines at a high concentration (25 mg/mL) can form a gel-like guanine tetramer structure [13]. A very stable structure was subsequently found in synthetic oligo-guanine nucleotides, which exhibited poor pairing capability with oligo-cytosine nucleotides, and X-ray crystal diffraction revealed that this construct was a higher-order structure formed by stacking guanine tetramer planes [14]. G4s are formed with two or more stacked G-quartets, which are square coplanar arrays of four guanine bases joined through eight Hoogsteen hydrogen bonds (Fig. 1B) [3, 15]. Owing to the physicochemical characteristics of the G-quartet, monovalent cations, such as K^+^, Na^+^ and NH_4_^+^, play important roles in the stabilization of G4s (Fig. 1C) [5, 16]. Moreover, depending on the number of G-quartet planes, the length of the connecting loop, the G-tract direction and the strand polarity, G4s exhibit polymorphisms consisting of parallel, antiparallel and hybrid structures [17–19]. In addition to in vitro evidence of G4 formation, G4 signals have also been detected in cells, such as U2OS cells, MCF-7 cells and HeLa cells (9). The number of G4 foci disappeared after DNase treatment but markedly increased when the cells were treated with the G4 stabilization ligand pyridostatin (PDS), confirming the specific recognition of G4s in cells by BG4 (9). In addition, with the help of G4-binding proteins, such as DEAH-box helicase 36 (DHX36) [20], LARK [21], pif1 [22] and the G4-specific antibodies BG4 [9] and 1H6 [23], G4s have been directly or indirectly detected in insect Bm12 cells (21) and human tissue cells, such as the skin, pancreas, testis and placenta (23). The presence of G4s in cells and tissues implies that these structures may participate in the regulation of important cellular activities.

Taking advantage of DNA sequencing and bioinformatics analysis, the prevalence of G4s has been reported in the genomes of many species. In the human genome, approximately 700,000 PQSs have been identified [24]. The number and abundance of PQSs in 37 species have increased with evolution [25]. Intriguingly, the distribution of G4s in genomes is not random but enriched in regulatory regions, such as promoters, replication origins, telomeres and UTRs, indicating their extraordinarily important biological functions [8]. Emerging evidence has confirmed that G4s participate in a series of fundamental cellular processes, such as DNA replication, gene transcription, telomere maintenance and RNA processing [26].

**Fig. 2:**
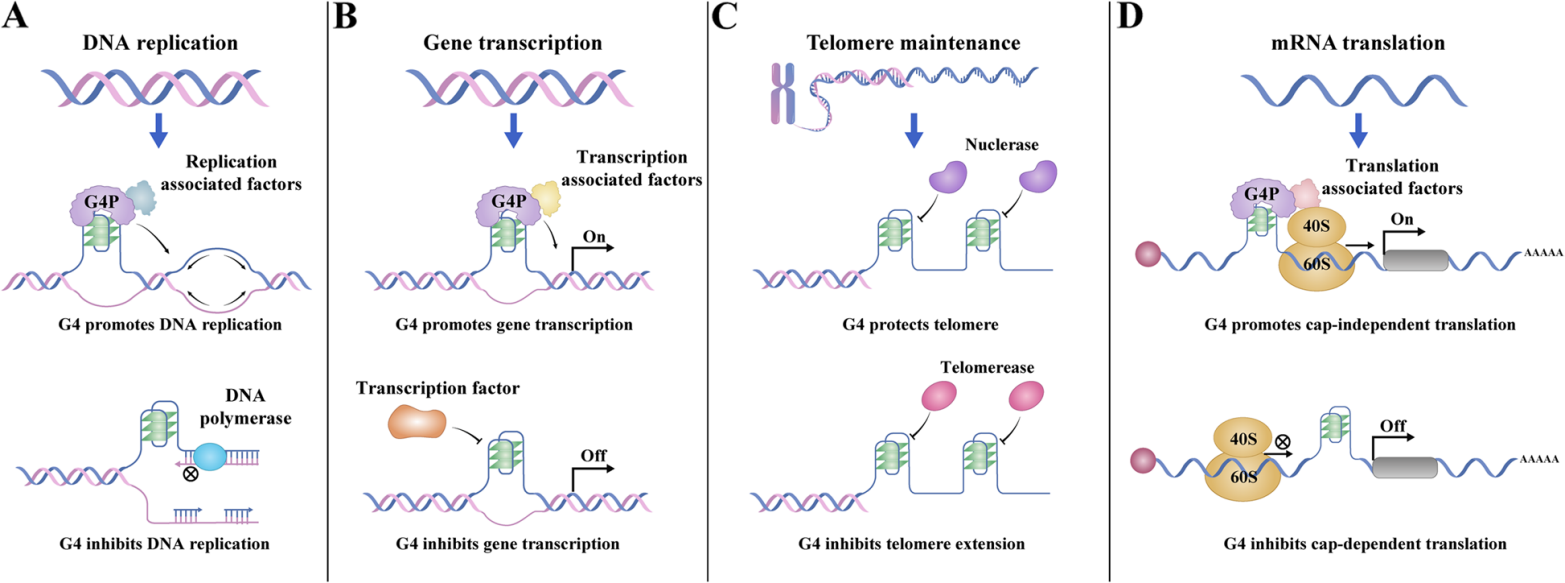
The biological functions of G4s. **A** G4s involved in DNA replication. G4s may function as presumed recognition sites to initiate replication through the recruitment of replication initiation factors or act as obstacles to inhibit DNA replication. **B** G4s in gene transcription. G4s can either promote or inhibit gene transcription by influencing the binding between the promoter and transcription factors or transcription initiation factors. **C** G4s involved in telomere maintenance. G4s function as inhibitors of telomerase activity to shorten telomeres or against nuclease activity to maintain telomere length. **D** RNA G4s in translation. RNA G4s can promote cap-independent translation by interacting with the 40S ribosome or inhibit translation by impeding the scanning of ribosomes

In mammalian genomes, 80%−90% of replication origins are GC rich and have the potential to form G4s [27–29], indicating the importance of G4s in DNA replication. Dual functions of G4s have been reported in DNA replication. G4s may act as obstacles to inhibit DNA replication, which requires helicases to unfold the G4s to avoid genome collapse and fork stagnation (Fig. 2A) [30, 31]. G4s may also function as presumed recognition sites to initiate replication through the recruitment of replication initiation factors, such as the origin recognition complex [32], Treslin–MTBP complex [33] and replication timing regulatory factor 1 (Rif1) [34] (Fig. 2A).

Importantly, G4s are abundant in promoter regions in various genomes. In the human genome, approximately 50% of human genes contain at least one PQS in their regulatory region [35]. Similarly, high levels of PQSs in promoter regions are found in the genomes of other mammals [36], yeasts [37], plants [38] and bacteria [39], suggesting a potential regulatory function of G4s in gene transcription. The first discovery of G4s affecting gene transcription was the human oncogene cellular myelocytomatosis (*c-Myc*), the expression of which was suppressed when cells were treated with the G4 ligand TMPyP4 [40, 41]. Furthermore, when *c-Myc* G4 was disrupted via the CRISPR-Cas9 gene editing method, the expression of *c-Myc* dramatically decreased, directly revealing the participation of G4s in the transcriptional regulation of *c-Myc* [42]. To date, several G4-regulated genes, especially oncogenes, such as v-kit Hardy-Zuckerman 4 feline sarcoma viral oncogene homolog (*KIT*), harvey rat sarcoma viral oncogene homolog (*HRAS*), vascular endothelial growth factor (*VEGF*), and B-cell lymphoma 2 (*BCL-2*), have been identified [43]. By using G4-binding proteins/oligands with the CRISPR-dCas9 system, specific G4s in a genome can be targeted to change the expression of the corresponding genes, by which the regulatory function of G4s in gene transcription can be demonstrated [44]. Recently, promoter G4s have been demonstrated to be common binding hubs for many different transcription factors to increase transcription [45]. Depending on the gene type and structure, G4s can either promote or inhibit gene transcription by influencing the binding between the promoter and transcription factors or transcription initiation factors (Fig. 2B).

Telomeres adopt abundant G4s because of the existence of a single-stranded G-rich 3ʹ overhang. The sequences of telomeres in Oxytricha, Tetrahymena and humans have been shown to be capable of forming G4s in vitro [46–48]. An antiparallel G4 was demonstrated in *Stylonychia lemnae* telomeres in vivo by using the specific G4 antibodies Sty49 and Sty3 [49]. Telomere G4s function as inhibitors of telomerase activity and telomere DNA replication to shorten telomeres or against nuclease activity to maintain telomere length (Fig. 2C) [50–52].

In addition to DNA G4s, guanine-rich RNAs can also form stable G4s. RNA G4s commonly contain two G-quartets and have parallel conformations [53]. RNA G4s play important roles in RNA processing, including translation, splicing, mRNA transport and mRNA stability (Fig. 2D) [26]. G4s within the 5’UTRs of mRNAs, such as fragile X mental retardation protein (*FMRP*), neuroblastoma RAS viral oncogene homolog (*NRAS*) and *BCL-2*, can inhibit translation in vitro, probably by influencing the recruitment of translation initiation factors or impeding the scanning of ribosomes [54–57]. G4s located in internal ribosome entry sites (IRES elements) within the 5’UTRs of the fibroblast growth factor 2 (*FGF2*) and *VEGF* mRNAs were found to promote cap-independent translation, probably by interacting with the 40S ribosome [58–60]. G4s formed near splice sites can act as exon splicing enhancers (e.g., in *FMRP*), intron splicing enhancers (e.g., in tumor protein 53, *TP53*) or silencers (e.g., in human telomerase reverse transcriptase, *hTERT*) to regulate alternative splicing [53]. In mouse neurons, RNA G4s are reportedly present in the 3’UTRs of calcium/calmodulin-dependent protein kinase II alpha (*CaMKlla*) and postsynaptic density protein 95 (*PSD-95*) mRNAs and act as neurite-targeting elements to affect the localization of these mRNAs in cortical neurites [61]. Recently, by comparing the nucleotide compositions of transcriptomes across 1,000 plants from different habitats, G-rich transcriptomes that tended to form RNA G4s were identified in plants growing in cold climates. Moreover, RNA G4s were found to be responsive to cold treatment in *Arabidopsis thaliana,* and these cold-responsive G4s served as mRNA stabilizers to increase the stability of mRNAs and participate in the regulation of plant cold-responsive growth [62]. Most recently, a strong positive correlation between G4 patterns in the region encoding 16S rRNA genes and the optimal growth temperatures of bacteria was revealed after analyzing 681 bacterial genomes. Evolutionary analysis revealed distinctive differences in G4 stability between *Thermotoga* (Topt ≥ 80 °C) and *Pseudothermotoga* (60 °C ≤ Topt < 80 °C) species, with *Thermotoga* species exhibiting greater G4 stability, suggesting that the G4 structures in 16S rRNA regions are key indicators of thermal adaptation in prokaryotes [63].

### G4s in plants

**Fig. 3:**
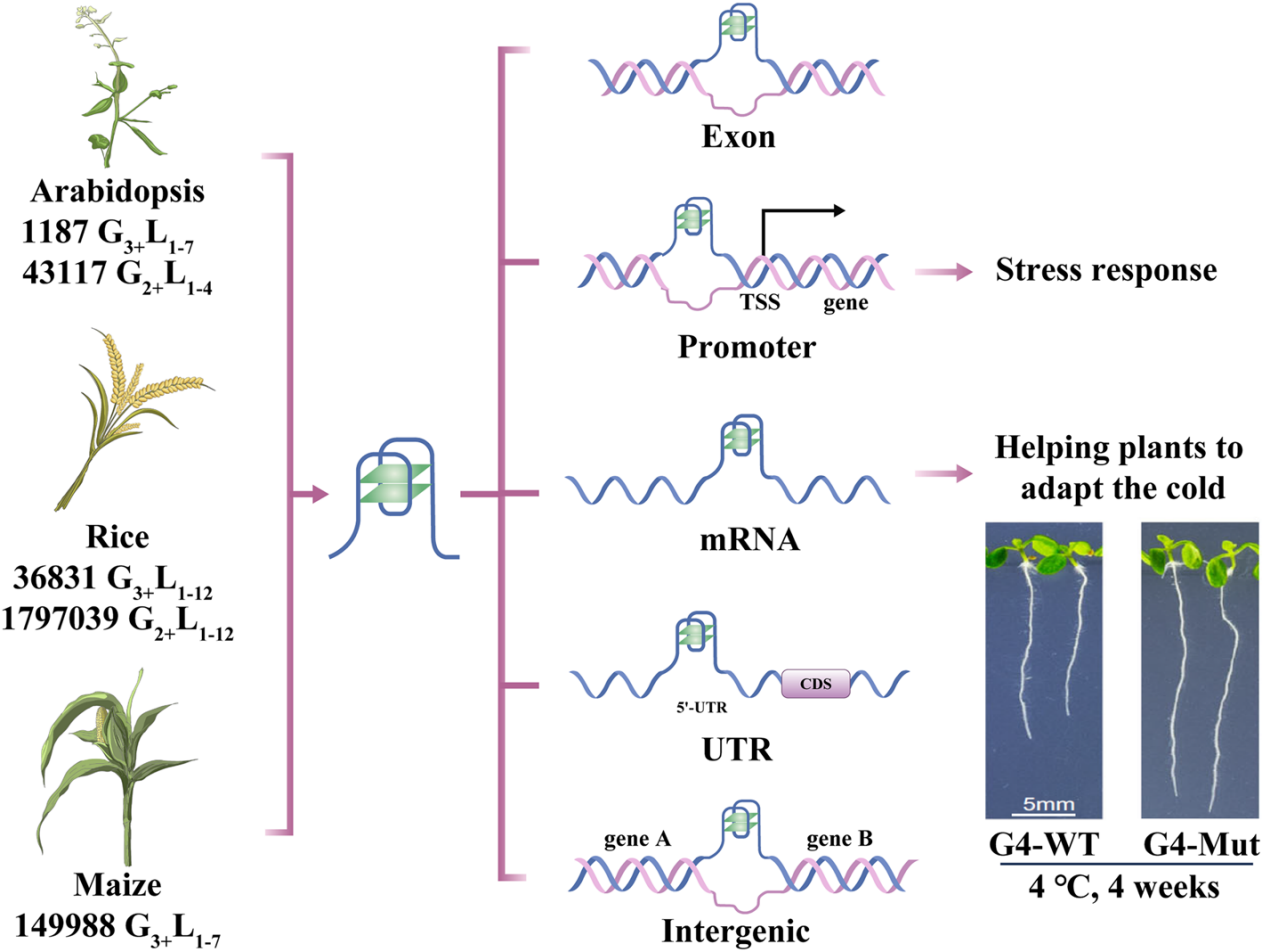
Distribution and functions of G4s in plants. In *Arabidopsis*, rice and maize, PQSs or G4s have been identified in exons, promoters, mRNAs, UTRs and intergenic regions. Promoter and mRNA G4s have been demonstrated to be associated with plant responses to stress, such as the response to cold climates. The RNA G4 could strongly increase mRNA stability and the response to cold climates by modulating plant root length. When RNA G4 was mutated, the root length of *Arabidopsis* was notably longer than that of the wild type at 4 °C

Although studies on plant G4s have increased only in recent years, evidence has revealed that G4s play vital functions in the regulation of plant development, especially under environmental stress, suggesting that G4s are potential targets for the development of improved crop varieties. Through genomic sequence analysis, the genome-wide distribution of PQSs has been studied in four plant species: *A. thaliana*, *Oryza sativa*, *Populus trichocarpa* and *Vitis vinifera* [64]. The PQSs in the genomes of these plants are enriched in the template strand at the transcription start site (TSS), suggesting potential *cis*-regulatory roles of G4s in gene transcription [64]. Genome-wide G4 analysis of 15 sequenced plant genomes, including those of *A. thaliana*, *O. sativa*, *Glycine max*, *Cicer arietinum*, *Medicago truncatula*, *Lotus japonicus*, *Phaseolus vulgaris*, *Brassica rapa*, *Setaria italica*, *Brachypodium distachyon*, *Sorghum bicolor*, *P. trichocarpa*, *V. vinifera*, *Selaginella moellendorffii* and *Physcomitrella patens*, revealed that the types of G4 enriched in the plant genomes were quite different from those in the human genome. In each of the plant species, G4s with two G-quartets (G2-type) were the most abundant (more than 90% of all G4s), and the percentage of G4s with three G-quartets (G3-type) was less than 5% [65]. Additionally, consistent with that in mammals, the genomic distribution of G4s in plants is not random but enriched in regulatory regions, such as those around the 5’UTR and TSS [66–68]. Genomic G4 analysis in 15 plant species revealed that G2-type G4s were enriched in regions closely associated with transcription and translation, such as genic, coding sequence (CDS), and exonic regions, whereas G3-type G4s were enriched in intergenic, promoter and intronic regions (Fig. 3) [65]. Genome-wide sequence analysis revealed that the PQS density in monocotyledons was five to ten times greater than that in dicotyledons [65, 67]. Intriguingly, PQSs were also predicted in chloroplast DNA and mitochondrial DNA, and the G4 frequency in mitochondrial DNA was approximately three times greater than that in nuclear DNA and chloroplast DNA [68]. The nonrandom location of G4s suggests their functional roles in plant development. In *A. thaliana*, RNA G4s were enriched in the genes responsible for drought and phenolic compound biosynthesis [69]. Gene Ontology (GO) analysis of the genes containing G4s in their template strand around the TSSs of the *O. sativa* genome revealed that chloroplast- and nucleosome-related GO terms, such as chloroplast photosystem I, histone acetylation, histone H3-H9 demethylation, histone binding and histone H3 acetylation, were enriched, indicating the probable functional role of G4s in chloroplasts [64]. In addition, G4s identified by the antibody-DNA immunoprecipitation (BG4-DNA-IP) method in maize were found to be closely associated with stress responses, such as hypoxia, oxidative stress and energy status [66]. Kwok et al. identified the first RNA G4 in the 5’ UTR of a DNA damage-responsive gene, ataxia telangiectasia-mutated and rad3-related (*ATR*), and reported that this G4 acted as a translational repressor in living plant cells [70]. Recently, Cho et al. reported that the RNA G4 form in the 5’UTR of the suppressor of max2 1-like4/5 (*SMXL4/5*) inhibited its translation and restricted phloem differentiation [71]. In addition, Yang et al. reported that the RNA PQS, which is located in the 3’ UTR of the homolog induced by drought 11 (*HIRD11*) gene encoding a KS-type dehydrin, could form G4s and inhibit their translation. When the Hird-11 mutant *A. thaliana* was complemented with *HIRD11* containing the wild-type RG4 sequence, the root length was short; however, the root length was significantly longer when RG4 was mutated, indicating that the G4 structure of HIRD11 inhibited its translation and further influenced the growth and development of *A. thaliana* [72]. Furthermore, the same author reported that the transcriptomes of plants growing at low temperatures presented more G4s, which may have helped the plants adapt to cold stress during evolution [62]. These studies highlight the biological role of G4s in plant development, especially in response to stress (Fig. 3). In addition, the tRNA-derived RNAs (tDRs) present in plants can form RG4s and bind with the DEA (D/H) RNA helicase (DExH1) to modulate accessible and functional tDRs to inhibit translation in plant cells [73]. Investigating the molecular mechanisms of G4 formation and regulation in plants, which may provide novel pathways and strategies for crop improvement, is highly interesting.

Since the enrichment of G4s in cancer tissues [74] and in promoters of many oncogenes, such as *c-MYC*, *VEGF*, kirsten rat sarcoma viral oncogene homolog (*k-RAS*) and *BCL-2* [43], G4s have been considered attractive targets for cancer therapies [12]. G4 ligands that can specifically bind with G4s have shown great potential for the development of anticancer drugs. In this context, plant-derived G4 ligands with low toxicity and easy availability have potential for cancer therapy. Several plant G4 ligands, such as flavonoids and alkaloids, have been identified [75]. Shang et al. successfully fished G4 ligands from natural plant extracts of *Phellodendron chinense Schneid cortexes* (PEs) via a combination of dialysis and the G4 recognition technique [76]. The dietary plant flavonoid fisetin strongly interacts with parallel G4s [77], and the flavonol kaempferol can specifically bind with VEGF G4 and increase its thermal stability [78]. The plant alkaloid chelerythrine can bind telomere G4s and G4s in the promoter of *c-MYC* [79, 80]. In addition, the alkaloid berberine, which has antitumour effects, can also recognize G4s [81]. Recently, several plant secondary metabolites, such as sanguinarine, quercetin, kaempferol and thymoquinone, were found to bind with *c-MYC* G4 and downregulate *c-MYC* expression [82]. Overall, screening natural plant G4 ligands is a promising strategy for the development of anticancer drugs, but more efforts are needed.

### G4s in insects

Insects are excellent model organisms for research because of their large populations, diverse species, and abundant genomic data resources. Insects are also ideal models for studying G4. In addition, as herbivores, insects play crucial roles in affecting crop growth and yield. In recent years, an increasing number of studies have revealed the functions of G4 in insects. Here, we reviewed the research progress on G4s from two perspectives: *Drosophila* and non-*Drosophila* insects.

### G4s in *Drosophila*

**Fig. 4:**
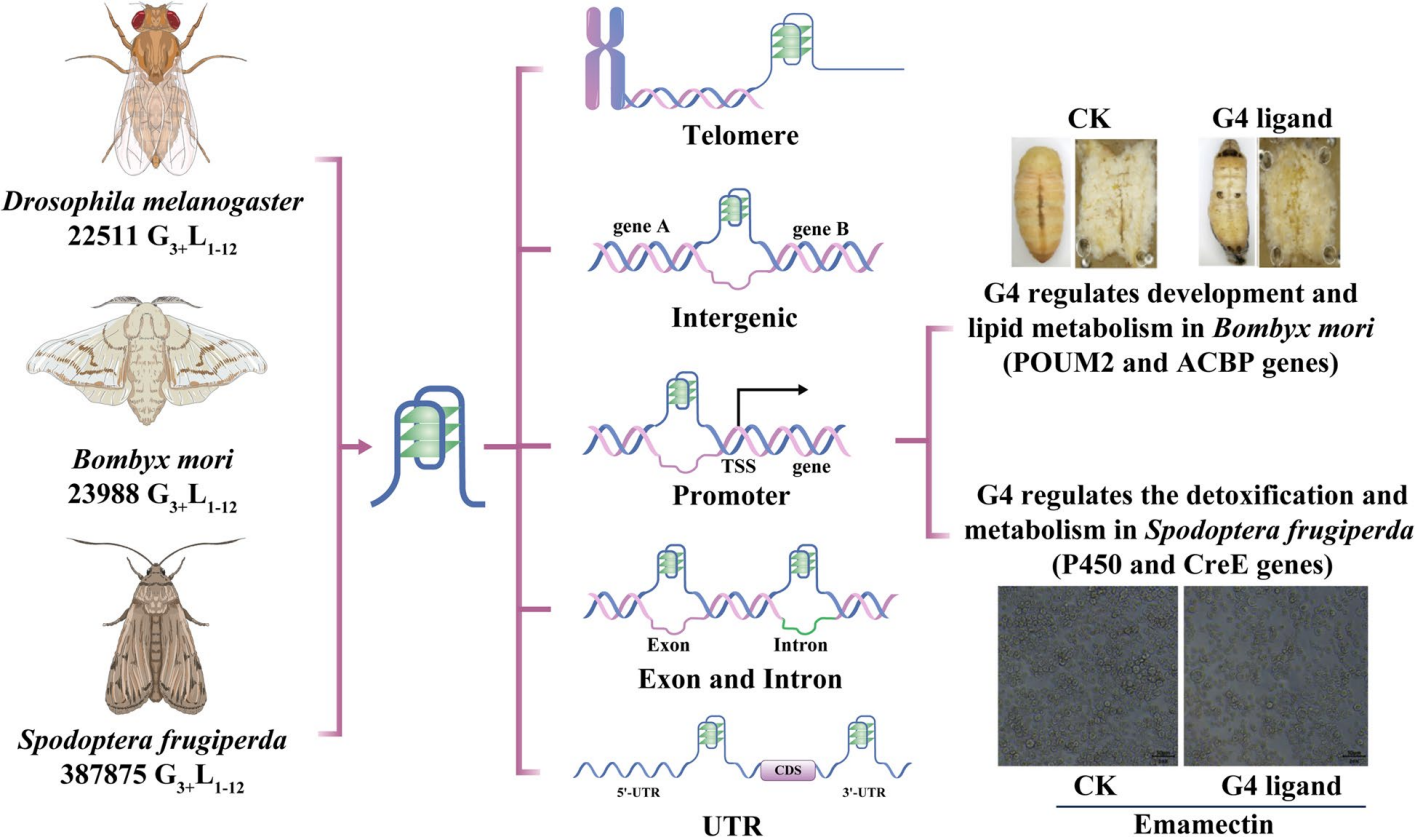
Distribution and functions of G4s in insects. In fruit flies, silkworms and fall armyworms, PQSs or G4s have been identified in telomeres, intergenic regions, promoters, exons, introns and UTRs. Promoter G4s have been reported to participate in the regulation of development and detoxification in insects. In silkworms, the expression of the development- and lipid metabolism-related genes BmPOUM2 and Bm ACBP is regulated by G4 in their promoter. Larvae treated with the G4 ligand PDS presented a reduction in fat body mass and a decrease in larval growth and metamorphic rates. In the fall armyworm, G4s were found to be involved in the regulation of detoxification genes, such as the P450 and CreE genes. G4 ligand treatment decreased the detoxication of sf9 cells, resulting in cell death

The first PQS identified in *Drosophila* was in the HeT-A element, which is a nonlong terminal repeat retrotransposon in *Drosophila* chromosome ends [83]. The conserved G-rich HeT-A elements in Chironomus and *D. melanogaster* play important roles in directing chromatin structure [84–86], highlighting the role of G4s in chromosome structure maintenance. G4s were then found to be associated with replication initiation because the PQSs significantly overlapped with the replication origins and were found to act as barriers to transiently stall the replication fork [87]. Furthermore, G4s were found to be common in all *D. melanogaster* centromeres [88]. By using an improved G4-seq method, a whole-genome experimental map of G4s in *Drosophila* was constructed, and 22,511 PQSs (G_3+_L_1–12_) were detected, 19,399 of which were enriched in lincRNAs, introns and promoters (Fig. 4) [89]. This *Drosophila* G4 map provides blueprints for G4-related studies in insects. G4 signals were detected in vivo in different organisms with the G4-specific antibody 1H6 [23]. In *Drosophila*, G4s have exquisite specificity for heterochromatin areas in polytene chromosomes of salivary glands, and the G4 signal is weaker in germline stem cells in *Drosophila* ovaries than in most other cells [90], implying a critical role of G4s in cell differentiation. In addition, in *Drosophila* expressing 30 G_4_C_2_ repeats, a key regulator of the nucleocytoplasmic transport RanGAP (*Drosophila* ortholog of human RanGAP1), which can physically interact with G_4_C_2_ repeats and suppress neurodegeneration, was identified [91]. Moreover, the well-known helicase DHX36 (also known as RHAU or G4R1) *Drosophila* homolog DmDHX36 was reported to bind with and unfold G4s [92]. The crystal structure of the DmDHX36 and G4 complexes was generated to reveal how DHX36 unfolds G4s [20]. Few but engaging studies on G4s in *Drosophila* have implied that G4s exist and have functions in *Drosophila*. On the basis of these findings, G4 ligands that can either stabilize or destabilize G4s (such as PDS) are regarded as new methods for pest control. Ligands that target G4s in either telomeres or centromeres could be used as a strategy to control fly survival by affecting chromosome maintenance. In addition, targeting G4-binding proteins could constitute another strategy for pest control. It has been reported that the G4-binding protein pif1 affects *Drosophila* embryo development, likely through influencing the resolution of G4 structures. In *Drosophila,* the pif1 mutation resulted in chromosome segregation defects, possibly due to the failure of G4 disruption, which stalls replication forks, leading to an accumulation of unresolvable replication intermediates [93].

### G4s in non-*Drosophila* insects

Although few studies on insect G4s have been conducted, increasing evidence suggests prominent roles for G4s in the regulation of insect growth and development (Fig. 4). The telomeric DNA of *Bombyx mori* was demonstrated to have the ability to form G4 structures in vitro via a combined nuclear magnetic resonance (NMR)‒molecular dynamics approach [94]. The *B. mori* telomeric d[TAGG(TTAGG)_3_] sequence, which is conserved in insects [95, 96], was reported to form a chair-type intramolecular G4 [97], and insect telomeric G4s are more stable in Na^+^ solution than in K^+^ solution [98]. The genome-wide characteristics of PQSs in 37 representative species ranging from fungi to humans revealed that the number, length and density of PQSs generally increased during evolution [25]. Immunofluorescence in seven species confirmed the presence and evolutionary pattern of G4s. G4s tended to be enriched in genetic regions, and the proportion of G4-bearing genes and orthologous genes also gradually increased with species evolution [25]. In the *B. mori* genome, 23,988 G4s have been predicted, most of which are located in intron, exon, intergenic and promoter regions [25], implying that G4s may participate in the regulation of gene transcription in insects. The first G4 gene shown to participate in transcriptional regulation in insects was identified in *B. mori* [21, 99]. This G4 was identified in the promoter of the *Bombyx mori* POU domain transcription factor 2 gene (*BmPOUM2*), which has been reported to regulate various developmental processes in insects [100, 101]. The G4-binding protein LARK (a homolog of RBM4 in humans) was identified and demonstrated to bind G4 in the *BmPOUM2* promoter to increase the transcription of *BmPOUM2* [18, 102]. In addition, by using the specific G4-binding protein LARK, G4 signals were also detected in the *B. mori* ovarian cell line Bm12 [21]. Knockout of LARK in *B. mori* via the CRISPR/Cas9 method resulted in embryonic lethality accompanied by significant changes in the expression of 63 putative cuticle protein genes, 8 wing disc cuticle protein genes and 5 pigment synthesis genes, indicating the functional importance of the G4- and G4-binding protein LARK in silkworm embryo development [103]. Furthermore, G4 has also been identified in the promoter region of the acyl-CoA binding protein (*ACBP*) gene in vertebrates and invertebrates and has been shown to bind with the LARK protein to regulate the transcription of *BmACBP*. When fifth-instar larvae were treated with the G4 ligand PDS, the expression of *BmACBP* and triacylglycerol decreased, revealing that G4s participate in lipid metabolism in silkworms and other species (Fig. 4) [104]. G4 was identified in the promoter of the silk gland factor-1 gene (*SGF1*) in *B. mori* and was demonstrated to regulate the expression of *BmSGF1* and silk protein production. When G4 was knocked out, the expression of *BmSGF1* in the posterior silk gland of *BmSGF1* G4 mutant larvae was notably reduced in the wandering stage when the silkworms started to transform and form cocoons, and silk production significantly decreased. These findings suggest that G4 acts as a positive regulator of *BmSGF1* transcription. [105]. Moreover, when larvae were treated with the G4 ligand PDS, the expression of *BmSGF1* was elevated [105].

In *Helicoverpa zea*, a G4 was identified to be located within the inserted transposon HzIS1-3 (known as *HzIS1-3* G4) in the promoter of the cytochrome P450 gene *CYP321A1*, which is a xenobiotic-metabolizing P450 gene responsible for plant allelochemicals and insecticides [106, 107]. *HzIS1-3* G4 could fold into an intramolecular G4 structure and act as a silencer to downregulate the constitutive and induced expression of *CYP321A1.* The destruction of *HzIS1-3* G4 or treatment with the G4 stabilizer N-methyl mesoporphyrin IX (NMM) resulted in reduced basal and flavone-/xanthotoxin-induced promoter activity of *CYP321A1* [108]. Recently, genome-wide G4 distribution analysis was conducted in the lepidopteran crop pest *Spodoptera frugiperda,* and 387,875 PQSs were identified, 66.9% of which were enriched in the upstream regions of start codons [109]. Notably, the genes containing PQSs in their promoter were enriched in metabolic pathways, especially the metabolism of xenobiotics by cytochrome P450 and drug metabolism. The expression and enzyme activity of P450 were significantly suppressed by treatment with the G4 ligand NMM, which increased the mortality of *S. frugiperda* (Fig. 4) [109]. These findings suggest that G4s are present in the genomes of lepidopteran insects and participate in the regulation of gene expression and development either as positive or negative regulators.

### Potential applications of G4s in pest control

Currently, the potential application of G4s as molecular targets for drug development mainly focuses on disease therapy, including various cancers and virus-mediated diseases [12]. Considerable evidence has shown that the number of G4s formed in the genome is significantly greater in tumor tissues than in normal tissues [74]. Moreover, G4s are enriched in the promoters of many oncogenes and have been reported to regulate the expression of oncogenes [43], indicating a close association between G4s and cancer and suggesting that G4s are promising new targets for anticancer strategies. Bioinformatic analysis revealed that the genomes of many viruses, including the prevalent human immunodeficiency virus (HIV) and severe acute respiratory syndrome coronavirus 2 (SARS-CoV-2) viruses, contain many PQSs [110, 111], and some of these PQSs can form G4s in vitro [112]. For example, a stable RNA, G4, was identified in the SARS-CoV-2 genome and could form in living cells, and the G4 ligand PDP could stabilize this RNA, G4, and significantly reduce the level of SARS-CoV-2 [113], indicating the potential for this G4 to be a novel target for antiviral therapy. Currently, G4 ligands, including G4-binding proteins and chemical compounds, have been screened for specific drugs that target the G4 structure of specific genes. Many compounds, such as the tetra-substituted naphthalene-diimide derivative (MM41), BRACO-19 and CX-5461, bind G4s and serve as antitumour drugs. MM41 was demonstrated to downregulate the transcription of *BCL2* and *KRAS* through binding and stabilizing the G4s in their promoters [114]. BRACO-19 is a type of telomere G4 stabilizer that binds G4s to inhibit telomerase activity, resulting in telomere shortening and end-to-end chromosomal fusion in cancer cells [115]. CX-5461, which has been in phase I clinical trials for BRCA1/2-deficient tumors, stabilizes G4s, leading to DNA damage and then genomic instability and the induction of apoptosis in tumor cells [116].

Similarly, because G4s in insects and plants participate in the regulation of gene expression and development, specific drugs that target G4s can be designed to modulate the expression of development-associated genes to control pest growth and development or to improve plant resistance. Many G4s have been found in stress-responsive genes in plants and in genes related to xenobiotic metabolism in insects [75, 103] (Table 1). In *B. mori*, G4 was demonstrated to promote the transcription of the wing disc development-related gene *BmPOUM2,* and knocking down this gene resulted in failure to complete metamorphosis [21, 101]. Additionally, BmACBP, a ubiquitously expressed protein responsible for lipid metabolism, was revealed to be regulated by G4 in its promoter, highlighting the function of G4s in lipid metabolism in insects. PDS treatment and knockout of G4 resulted in reductions in fat body mass and suppressed larval growth and metamorphic rates [104]. In response to toxic compounds, including phytochemicals and pesticides, insects have evolved efficient detoxification enzyme systems to resist toxic compounds, such as cytochrome P450-dependent monooxygenases (P450s), glutathione-*S*-transferases (GSTs), and carboxylesterases (COEs). A genome-wide analysis of G4 in the genome of *S. frugiperda*, a globally distributed lepidopteran crop pest, revealed that G4s were enriched in the promoter regions of the P450 and CarE genes and that treatment with the G4 ligand NMM downregulated the expression of P450 genes, such as *CYP9A58*, *CYP9A60*, *CYP6B2*, *CYP6B6*, *CYP9A21v3*, *CYP4C1*, *CYP4C1*, *CYP49A1* and *CYP18A*, and *CarE3*, synergizing with the effects of insecticides [109]. Currently, G4 is widely accepted as a novel epigenetic regulatory mechanism of the transcription of genes that are associated with the development of organisms. During evolution, the number and distribution density of G4s in the genome have increased exclusively from lower to higher organisms, indicating that G4s may become an evolutionary regulatory mechanism to enable higher organisms to achieve more complex behaviors, such as adapting to environmental changes [25]. Pečinka et al*.* compared the G4 content of three gene sets from *A. thaliana*, including those related to drought stress-responsive genes, the production or biosynthesis of phenolic compound-related genes and housekeeping genes, and reported that the highest number and frequency of PQS in the phenolic compound biosynthesis gene set were associated with the *TT5* gene, which is related to flabvanone production [69]. *TT5* encodes a chalcone‒flavanone isomerase family protein that catalyzes the conversion of chalcone into flavanone. Flavanone is a phenolic compound that participates in plant defense against pests [117].

**Table 1:** List of functional studies on G4s in plants and insects

Species	G4 location	Molecular function	Biological function	References
*Arabidopsis thaliana*	5’UTR of *SMXL4/5*	Translation suppression	Affect the phloem differentiation	[71]
*Arabidopsis thaliana*	3’UTR of *HIRD11*	Translation suppression	Regulate the plant root growth	[72]
*Arabidopsis thaliana*	3’UTR of *AT1G13390*	mRNA stabilization	Facilitate plants to adapt to cold climate	[62]
*Arabidopsis thaliana*	tRNA-derived RNA(tDRs)	Translation inhibition	Modulate accessible and function tDRs	[73]
*Drosophila melanogaster*	Telomere	Telomere protection	Chromosome maintenance	[83]
*Drosophila melanogaster*	Centromere	Centromere specication	Ensure the accurate inheritance of genetic information	[88]
*Bombyx mori*	Promoter of *POUM2*, *ACBP* and *SGF1*	Transcription activation	Modulate the transcription of critical developmental genes	[21, 104 105]
*Bombyx mori*	Telomere	Telomere protection	Chromosome maintenance	[94, 97]
*Helicoverpa zea*	Transpson in the promoter of *CYP321A1*	Transcription suppression	Inhibition of detoxification metabolism	[108]
*Spodoptera frugiperda*	Promoter of *P450*, *CreE* genes	Transcription suppression	Inhibition of detoxification metabolism	[109]

**Fig. 5:**
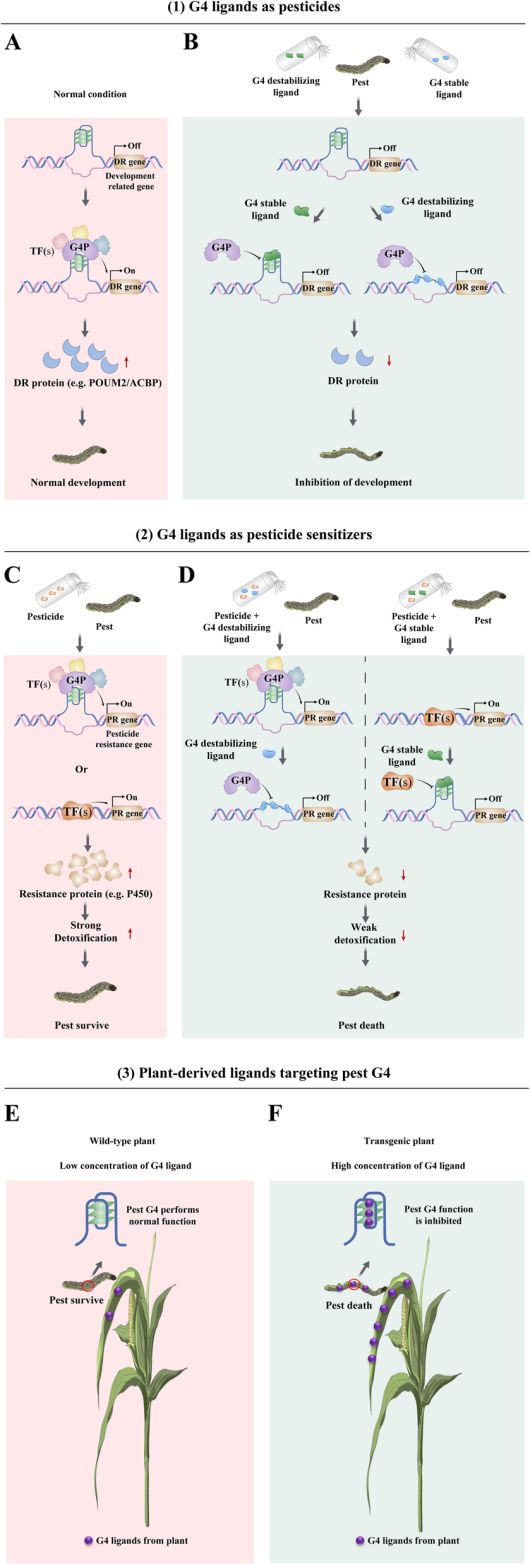
Three potential strategies for pest control involving the targeting of G4s. **A** Under normal conditions, G4s regulate the transcription of development-related genes (DR genes), such as *BmPOUM2* and *BmACBP*, by recruiting G4-binding protein (G4P) and cotranscription factors (TFs). **B** When pests are treated with G4-stable or destabilizing ligands, these G4 ligands inhibit the recruitment of G4P, thereby affecting the transcription of DR genes and affecting the normal growth and development of the pests. **C** When only pesticides are used, pests can reduce their sensitivity to pesticides by upregulating the expression of detoxification enzymes, such as P450 and CarE, through the use of G4s or the normal regulatory method. **D** The combined use of pesticides and G4-destabilizing ligands or G4-stable ligands can increase the sensitivity of pests to pesticides by inhibiting the formation of G4s or by inhibiting the binding of G4-binding protein (G4P) to G4s. **E** Under normal conditions, the concentration of G4 ligands in plants is relatively low, which is not enough to affect the growth and development of pests by binding to pest G4. **F** The concentration of G4 ligands in plants can be increased by constructing transgenic plants. When insects feed on these plants, high concentrations of G4 ligands affect G4 function, thereby affecting the growth and development of the pests

Owing to differences in G4-forming sequences, G4 structures exhibit structural specificity. We can achieve specific regulation of a gene by screening ligands that are specific to its G4 structure. For pest control, we need to focus on and thoroughly investigate the structural characteristics and functions of important G4s of critical genes (such as development or pesticide resistance genes). Thus, possible pest control strategies can be proposed. 1) G4 ligands, which can enter cells to stabilize or destabilize G4s, can be used as pesticides by targeting the G4 structures of important development-related genes. These ligands can interfere with the expression of these genes, resulting in abnormal larvae (Fig. 5A, B). 2) G4 ligands can be used as pesticide sensitizers. When a pesticide is used alone, constant pesticide stimulation induces the upregulation of detoxification enzyme-encoding genes, such as P450, GSTs, and COEs, increasing resistance to pesticides (Fig. 5C). When a pesticide is used along with a G4-destabilizing ligand, the G4 ligand binds to and disrupts G4, resulting in the suppression of detoxification enzyme-encoding genes, thus strengthening the insecticidal effects (Fig. 5D). In other cases, when a pesticide is applied along with a stable G4 ligand, the G4 ligand binds to G4 to form a G4-ligand complex, which inhibits the binding of transcription factors and therefore reduces the expression of detoxification enzyme-encoding genes, synergizing with the efficiency of the insecticide (Fig. 5D). 3) Plant-derived ligands can target pest G4s. It is believed that many G4 ligands exist in plant extracts, from which we can screen specific compounds that target pest G4s. The concentration of G4 ligands in plants can be increased by constructing transgenic plants. Thus, when pests feed on these plants, the host ligands can bind with G4s in the pests and influence the transcription and function of corresponding genes, disturbing the development of the pests (Fig. 5E, F). A promising method for screening natural G4 ligands from plants has been reported [71–77]. Plant G4 ligands are usually safer, have low toxicity and are easily obtained. The development of insecticides or synergists with plant-derived G4 ligands is an attractive strategy that is specific and environmentally friendly.

### Prospects

In this mini-review, we summarize new progress in the study of G4s in plants and insects. Although few studies have been reported, novel progress has emerged in recent years. The proven functions of G4s in the regulation of gene transcription and individual development provide potential for their application in pest control. Although no significant progress in the testing and application of G4-related compounds has been made, large-scale screening for G4-binding compounds from plants has been initiated. With more G4s identified and their regulatory functions confirmed in insects and plants, novel insecticides and strategies targeting DNA or RNA G4s may provide a new potential direction for pest control. Unlike currently used insecticides, which target either a physiological process, such as organophosphorus pesticides, or signal transduction, such as hormone analogs, G4-associated insecticides specifically target a G4 of a DNA or RNA molecule, which is likely more species-specific and environmentally friendly.

## Data Availability

Not applicable.
